# An Improved Calibration Technique for MEMS Accelerometer-Based Inclinometers

**DOI:** 10.3390/s20020452

**Published:** 2020-01-13

**Authors:** Jiaxin Zhu, Weifeng Wang, Shiping Huang, Wei Ding

**Affiliations:** 1 School of Civil Engineering and Transportation, South China University of Technology, Guangzhou 510640, China; ctjxzhu@mail.scut.edu.cn (J.Z.); ctwfwang@scut.edu.cn (W.W.); ctwding@mail.scut.edu.cn (W.D.); 2 State Key Laboratory of Subtropical Building Science, South China University of Technology, Guangzhou 510640, China

**Keywords:** calibration, micro-electro-mechanical system accelerometer, inclinometer, deformation

## Abstract

Micro-electro-mechanical system (MEMS) accelerometer-based inclinometers are widely used to measure deformations of civil structures. To further improve the measurement accuracy, a new calibration technique was proposed in this paper. First, a single-parameter calibration model was constructed to obtain accurate angles. Then, an image-processing-based method was designed to obtain the key parameter for the calibration model. An ADXL355 accelerometer-based inclinometer was calibrated to evaluate the feasibility of the technique. In this validation experiment, the technique was proven to be reliable and robust. Finally, to evaluate the performance of the technique, the calibrated MEMS inclinometer was used to measure the deflections of a scale beam model. The experimental results demonstrate that the proposed technique can yield accurate deformation measurements for MEMS inclinometers.

## 1. Introduction

Due to their low cost, small size, high durability, low power consumption, and easy installation [1], micro-electro-mechanical system (MEMS) accelerometer-based inclinometers have been widely applied in civil engineering, such as for deformation measurements of bridges [2] and buildings [3], for soil stability monitoring of embankments [4] and landslides [5], and for performance evaluations of underground structures [6]. In these cases, MEMS inclinometers are used to measure both angular deformations and displacements. With the increasing concern regarding structural safety, higher performances are required for MEMS inclinometers. Since MEMS inclinometers that are based on accelerometers have the potential to offer superior results with lower cost and higher stability, in this paper, we focus on improving the performance of MEMS accelerometer-based inclinometers.

In applications of MEMS inclinometers, many challenges have been encountered on the sensing module, which causes relatively inaccuracy in the measurement of small deformations of civil structures. The challenges mainly come from error effects of the offset, sensitivity mismatch, temperature effect, and noise [7,8,9]. Among these, the error effect of noise can be reduced by the average method effectively [10]. Therefore, general calibration methods mainly focus on the following error sources: (1) Offset, (2) sensitivity mismatch, and (3) temperature effect. The error effect of the offset refers to the deviation of the output of the sensors under a zero-g condition. The error effect of the sensitivity mismatch consists of the errors caused by axis sensitivity and cross-axis sensitivity. Axis sensitivity refers to the ratio between the input value and the output value of the accelerometer, while cross-axis sensitivity is a coupling coefficient between the output value of the measurement axis and the output values of other axes. The error effect of the sensitivity mismatch of MEMS inclinometers is mainly caused by manufacturing errors, unstable supply voltages, and temperature effects. The temperature effect causes the change of offset and sensitivity during measurements, which leads to errors. Consequently, many techniques have been proposed to reduce the errors mentioned above. Among them, calibration is proven to be the most effective method. In practical engineering, calibration is a required procedure for ensuring cogent measurement results.

Numerous studies have focused on mathematical models for calibration of the MEMS accelerometer-based inclinometer [11]. Many of these mathematical models rely on the result that under static conditions, accelerations measured by accelerometers should be equal in magnitude to the local gravitational acceleration. Based on this, they estimate parameters that are related to the scale factor, offset, nonlinearity, cross-axis sensitivity, and misalignment and use the parameters for calibration. Combining data from inclinometers, rate gyros, and accelerometers, Leavitt [12] proposed a linear time-invariant system that uses low-cost sensors to obtain precise angular measurements. Luczak [13] used a novel method that overcame the problem of nonlinearity to determine the tilt angles over the full measurement range accurately. Ang [14] identified the features of errors that are associated with the scale factor, offset, and misalignment of the dual-axis accelerometer and proposed a nonlinear regression model for reducing these errors. Parsa [15] used the least-squares method to calibrate the axis misalignment errors of MEMS accelerometers. Frosio [16,17] proposed a field calibration procedure for triaxial accelerometers and used the classical quadratic cost function to identify the optimal parameters of a linear sensor model of a MEMS accelerometer. Won [18] proposed a low-cost computational calibration method for MEMS accelerometers that includes six calibration parameters. Qian [19] proposed a least-squares-based linear model for accurately measuring tilts. Hu [20] presented a twelve-parameter model for calibrating uniaxial micro-accelerometers.

Most calibration methods either required expensive devices or involved complicated multiparameter procedures. In these calibration methods [18,20], sensors have to be placed in specific orientations, which is hard to practice. For example, it is difficult to find a flat plane to place the axis of MESE accelerometers at ±1 g field. Hence, the calibration methods are hard to satisfy frequent and repetitive calibrations in practical measurements. Moreover, as micro-electro-mechanical system technology further develops, the insufficient accuracy for small-deformation measurements mainly originates from the offset of accelerometers [21]. As shown in Table 1 [22], the error effects of temperature are only ±0.01%/°C to sensitivity and ±0.5 mg/°C to offset. Since most MEMS sensors have internal temperature sensors and the datasheets from manufacturers provide the relationships between error effects (offset and sensitivity) and the temperature effects, the errors caused by the temperature effect can be compensated during the measurement. Furthermore, due to the advanced manufacturing technology and available stable voltage power supply, the error effect of the sensitivity mismatch has been well reduced. More importantly, for structural deformation measurement, the error effect of the offset is more significant. For example, an offset of 50 mg can cause a measurement error of 3.440° (see Table 1). Therefore, a simple and accurate onsite calibration method for civil structures is needed.

To this end, an improved calibration technique is proposed in this work. In this technique, we constructed a mathematical model that contains only one parameter for calibrating relative angles directly. Furthermore, an image-processing-based method was designed to obtain the parameter value. Finally, validation and application experiments were conducted to evaluate the feasibility and performance of the proposed technique. 

The remainder of this paper is organized as follows: Section 2 demonstrates the improved calibration technique. Section 3 presents and discusses the validation experiments. Section 4 presents an application experiment. The conclusions of this study are drawn in Section 5.

## 2. Single-Parameter Calibration Technique

### 2.1. Angle Sensing Principles

MEMS accelerometer-based inclinometers determine angles using a gravity vector and its projection on the accelerometer axes. For commonly used accelerometers, three types of algorithms are available for the conversion from accelerations to angles: (1) An algorithm that is based on the inverse tangent function, (2) an algorithm that is based on the inverse sine function, and (3) an algorithm that is based on the inverse cosine function. In this paper, the inverse-tangent-function-based algorithm is used because it is more accurate and requires less computation than other algorithms and has a constant effective incremental sensitivity [31,32]. 

The inverse-tangent-function-based algorithm uses Equations (1)–(3) to calculate the angles:

(1) $$\theta =\tan^{-1}\frac{A_{x,ot}}{\sqrt{A_{y,ot}^{2}+A_{z,ot}^{2}}},$$

(2) $$\psi =\tan^{-1}\frac{A_{y,ot}}{\sqrt{A_{x,ot}^{2}+A_{z,ot}^{2}}},$$

(3) $$\phi =\tan^{-1}\frac{\sqrt{A_{x,ot}^{2}+A_{y,ot}^{2}}}{A_{z,ot}},$$

**Figure 1:**
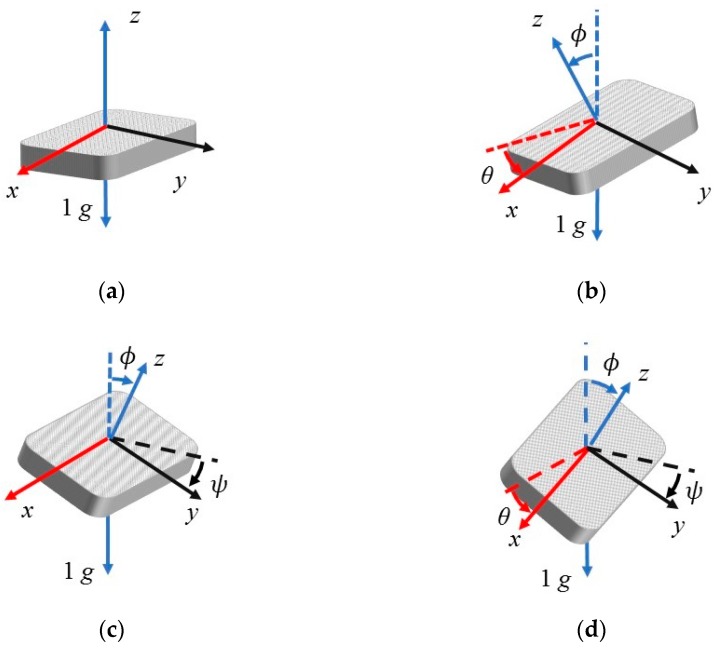
Sensing angles of the MEMS inclinometer. (**a**) The typical reference position; (**b**) an illustration of angles *ϕ* and *θ* and the relationship between them; (**c**) an illustration of angles *ψ* and *ϕ* and the relationship between them; (**d**) three sensing angles of the MEMS inclinometer.

In the algorithm, the angle of each axis of the accelerometer is determined separately from the reference position. The typical direction of the accelerometer (the x-axis and y-axis are in the 0 g field) is used as the reference position (see Figure 1a). *θ* is the angle between the horizontal line and the x-axis of the device, *ψ* is the angle between the horizontal line and the y-axis of the device, and *ϕ* is the angle between the gravity vector and the z-axis (see Figure 1b–d). *θ*, *ψ,* and *ϕ* are specified in degree. $A_{x,ot}$, $A_{y,ot}$, and $A_{z,ot}$ are outputs of the accelerometer, which are specified in units of mg. The inversion of Equation (3) is due to the reference position of the accelerometer (see Figure 1a). When the device is in the typical reference position, all measurement angles are 0° [21].

**Figure 2:**
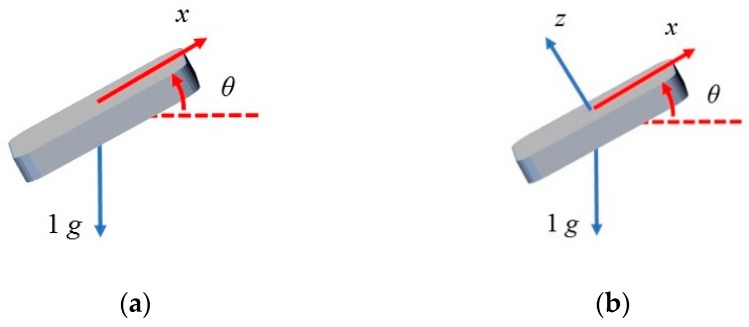
Sensing angles of the 1-axis and 2-axis MEMS accelerometer. (**a**) The 1-axis MEMS accelerometer; (**b**) the 2-axis MEMS accelerometer.

The equations above are derived based on 3-axis accelerometers, which have been widely used. However, they are also suitable for 1-axis and 2-axis accelerometers. Figure 2a illustrates the angular measurement of the 1-axis MEMS accelerometer, which is similar to the angular measurement of the angle *θ* of the 3-axis MEMS accelerometer, as seen in Figure 1b. The difference between the 1-axis MEMS accelerometer and the 3-axis MEMS accelerometer is that the former cannot read the data of the y-axis and z-axis. Therefore, for the 1-axis MEMS accelerometers, assuming $A_{y,ot}$ is equal to 0 mg and $A_{z,ot}$ is equal to $-\sqrt{{\left(1000\;mg\right)}^{2}-A_{x,ot}^{2}}$, the inverse-tangent-function-based algorithm can be applied. Figure 2b illustrates the angular measurement of the 2-axis MEMS accelerometer, which is also similar to Figure 1b. The difference between the 2-axis MEMS accelerometer and the 3-axis MEMS accelerometer is that the former cannot read data of the y-axis. By assuming $A_{y,ot}$ is equal to 0 mg, the algorithm can be applied to the 2-axis MEMS accelerometers.

### 2.2. Single-Parameter Mathematical Model

In contrast to the traditional calibration methods, which aim at compensating accelerations for angle calibration, the proposed technique calibrates relative angles directly by using an offset-related parameter. 

Since the deformation range is small for civil structures (−5 to +5° can satisfy the requirements), the linear approximation assumption can be applied in the inverse-tangent-function-based algorithm. Generally, only relative angles Δθ and Δψ are considered in applications of civil engineering. Thus, a conversion algorithm that considers the offset errors can be expressed as follows:

(4) $$\theta =\tan^{-1}\frac{A_{x,act}+A_{x,off}}{\sqrt{{\left(A_{y,act}+A_{y,off}\right)}^{2}+{\left(A_{z,act}+A_{z,off}\right)}^{2}}},$$

(5) $$\psi =\tan^{-1}\frac{A_{y,act}+A_{y,off}}{\sqrt{{\left(A_{x,act}+A_{x,off}\right)}^{2}+{\left(A_{z,act}+A_{z,off}\right)}^{2}}},$$

In these equations, $A_{x,ot}=A_{x,act}+A_{x,off}$, $A_{y,ot}=A_{y,act}+A_{y,off}$, and $A_{z,ot}=A_{z,act}+A_{z,off}$, where $A_{x,act}$, $A_{y,act}$, and $A_{z,act}$ are the actual outputs of the accelerometer in *mg*, and $A_{x,off}$, $A_{y,off}$, and $A_{z,off}$ are offsets of the accelerations in mg.

Since Equations (4) and (5) exhibit similarities, this paper only discusses *θ*. In the measurement range, using the linear approximation of the inverse tangent function, Equation (4) can be simplified to:

(6) $$\theta =\frac{A_{x,act}+A_{x,off}}{\sqrt{{\left(A_{y,act}+A_{y,off}\right)}^{2}+{\left(A_{z,act}+A_{z,off}\right)}^{2}}},$$

The logarithm of Equation (6) is expressed as follows:

(7) $$\ln\left|\theta \right|=\ln\left|A_{x,act}+A_{x,off}\right|-\ln\sqrt{{\left(A_{y,act}+A_{y,off}\right)}^{2}+{\left(A_{z,act}+A_{z,off}\right)}^{2}},$$

where $\sqrt{{\left(A_{y,act}+A_{y,off}\right)}^{2}+{\left(A_{z,act}+A_{z,off}\right)}^{2}}=\sqrt{A_{y,act}^{2}+A_{z,act}^{2}+2A_{y,act}A_{y,off}+2A_{z,act}A_{z,off}+A_{y,off}^{2}+A_{z,off}^{2}}$.

Under the typical reference position (see Figure 1a), we have the relationship that $A_{y,act}^{2}+A_{z,act}^{2}\gg 2A_{y,act}A_{y,off}+2A_{z,act}A_{z,off}+A_{y,off}^{2}+A_{z,off}^{2}$ within the measurement range. Thus, based on the linear approximation of the natural logarithmic function,$\mathrm{when}\;x\gg \Delta x,\;\ln\left(x+\Delta x\right)=\ln x+\frac{\Delta x}{x}$, Equation (7) can be simplified to the following equation:

(8) $$\ln\left|\theta \right|=\ln\frac{\left|A_{x,act}+A_{x,off}\right|}{\sqrt{A_{y,act}^{2}+A_{z,act}^{2}}}+\frac{2A_{y,act}A_{y,off}+2A_{z,act}A_{z,off}+A_{y,off}^{2}+A_{z,off}^{2}}{-2\left(A_{y,act}^{2}+A_{z,act}^{2}\right)},$$

By using the exponential function, Equation (8) can be expressed as follows:

(9) $$\theta =c\frac{A_{x,act}+A_{x,off}}{\sqrt{A_{y,act}^{2}+A_{z,act}^{2}}},$$

where $c=e^{\frac{2A_{y,act}A_{y,off}+2A_{z,act}A_{z,off}+A_{y,off}^{2}+A_{z,off}^{2}}{-2\left(A_{y,act}^{2}+A_{z,act}^{2}\right)}}$.

In the typical orientation and within the measurement range, $A_{x,act}$ and $A_{y,act}$ are small and $A_{z,act}$ is approximately 1000 mg, which is substantially larger than the other actual output values. Thus, $c\approx e^{\frac{-A_{z,off}}{1000\;mg}}$. By using the linear approximation, Equation (9) can be simplified to:

(10) $$\theta =e^{\frac{-A_{z,off}}{1000\;mg}}\left(\tan^{-1}\frac{A_{x,act}}{\sqrt{A_{y,act}^{2}+A_{z,act}^{2}}}+\frac{A_{x,off}}{1000\;mg}\right),$$

Therefore, the actual relative angles $\Delta \theta_{act}$ can be expressed as follows:

(11) $$\Delta \theta_{act}=e^{\frac{A_{z,off}}{1000\;mg}}\Delta \theta_{err}=e^{\frac{A_{z,off}}{1000\;mg}}\left(\theta_{1}-\theta_{2}\right),$$

By using the same manner, the actual relative angles $\Delta \psi_{act}$ are obtained as follows:

(12) $$\Delta \psi_{act}=e^{\frac{A_{z,off}}{1000\;mg}}\Delta \psi_{err}=e^{\frac{A_{z,off}}{1000\;mg}}\left(\psi_{1}-\psi_{2}\right),$$

In these equations, $\theta_{1}$ and $\psi_{1}$ are the initial angles, $\theta_{2}$ and $\psi_{2}$ are the updated angles after deformation, and $\Delta \psi_{act}$ are the actual relative angles without errors, and $\Delta \theta_{err}$ and $\Delta \psi_{err}$ are the raw relative $\Delta \theta_{act}$ angles that contain errors. According to Equations (11) and (12), having one key parameter, namely, $A_{z,off}$, can satisfy the calibration requirements for relative angles.

### 2.3. Method for Obtaining the Key Parameter 

**Figure 3:**
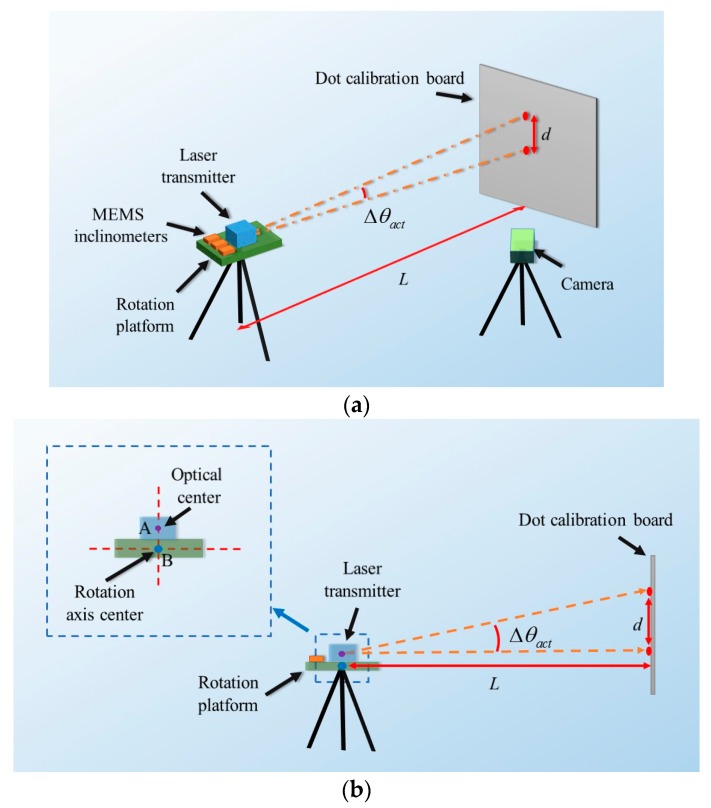
The proposed method for obtaining the key parameter. (**a**) Illustration of the method; (**b**) illustration of parameters of the method.

The key parameter $A_{z,off}$ of the model is essential, and we designed an image-based method for obtaining it. The image processing method has advantages of cost-effective, easy to implement, and highly accurate up to sub-pixel level [33,34]. In this method, a laser transmitter, a digital camera, a laser rangefinder, and a dot calibration board are needed. As illustrated in Figure 3, multiple MEMS inclinometers and a laser transmitter are mounted on the rotation platform of a tripod stably and horizontally. The optical center A of the laser transmitter is placed on the same vertical plane as the rotation axis center B of the rotation platform, and this plane is perpendicular to the rotation platform, as seen in Figure 3b. Rotating the platform with an actual relative angle $\Delta \theta_{act}$, the MEMS inclinometers mounted on the platform will output raw relative angles $\Delta \theta_{err}$, and the laser spot on the calibration board will move vertically for a distance *d*. *d* is the distance between the center point of the laser spot before the rotation and the center point of the laser spot after the rotation. Parameter *L* in Figure 3 refers to the distance between the rotation axis center B of the platform (in Figure 3b) and the calibration board. It is noted that the values of parameter *L* and parameter *d* are the same for all the MEMS inclinometers (for calibration) mounted on the same rotation platform, while the raw relative angles $\Delta \theta_{err}$ of each MEMS inclinometer are not the same. Thus, using *L*, *d,* and the corresponding raw relative angles $\Delta \theta_{err}$ of each MEMS inclinometer, the corresponding calibration parameter $A_{z,off}$ can be calculated for each MEMS inclinometer on the platform, as follows:

(13) $$A_{z,off}=1000\;mg\ln\frac{\Delta \theta_{act}}{\Delta \theta_{err}}=1000\;mg\ln\frac{\tan^{-1}\frac{d}{L}}{\Delta \theta_{err}},$$

**Figure 4:**
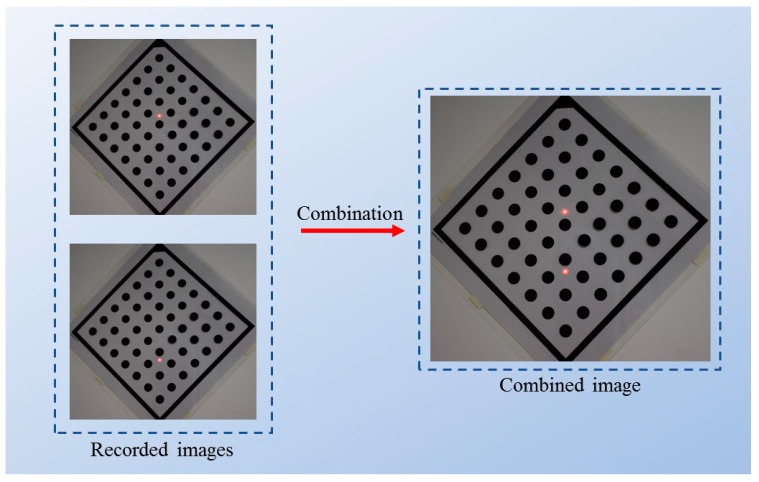
Combination of the recorded images.

It can be seen that for obtaining the parameter $A_{z,off}$, the parameters *d* and *L* are needed to be measured first. The vertical movement *d* is accurately measured via an image processing method. First, the camera is fixed and is used to record images with the laser spot (see Figure 3a), so all the images have the same coordinate systems. Therefore, the recorded images can be accurately assembled into a combined image for calculating *d* (see Figure 4). There are two processes, namely, detection and conversion, for calculating the vertical movement *d*.

**Figure 5:**
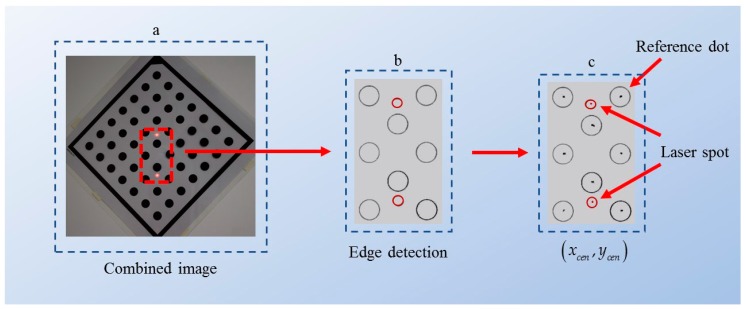
The process of detection. (**a**) The combined image sample; (**b**) edge detection; (**c**) centrioid coordinate calculation.

In the process of detection, it aims to identify pixel centroid coordinates for each object of interest in the combined image. As shown in Figure 5, a detection method [35,36] is used to detect the edge of each reference dot and the edge of each laser spot in the combined image (see Figure 5b). Each edge contains a set of discrete points, of which the pixel coordinates are denoted as array(x,y). Then, by using Equations (14) and (15), the pixel centroid coordinates $\left(x_{cen},y_{cen}\right)$ of the reference dots or the laser spots can be calculated [37], as shown in Figure 5c. Thus, the pixel distances between objects of interest can be calculated through the pixel centroid coordinates.

(14) $$x_{cen}=\frac{\sum_{x,y}^{}\left(array\left(x,y\right)\cdot x\right)}{\sum_{x,y}^{}array\left(x,y\right)},$$

(15) $$y_{cen}=\frac{\sum_{x,y}^{}\left(array\left(x,y\right)\cdot y\right)}{\sum_{x,y}^{}array\left(x,y\right)},$$

The process of conversion is used to calculate the actual length of vertical movement *d* from its pixel length $d_{pixel}$ by using the scale *s* (the ratio of the actual length to the pixel length), seen in Equation (16).

(16) $$d=sd_{pixel},$$

**Figure 6:**
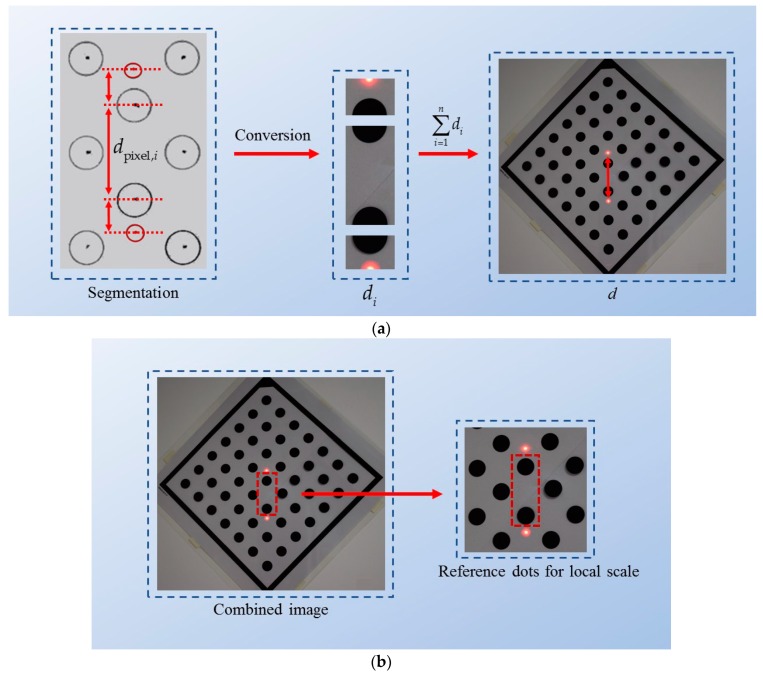
The process of conversion: Segmentation method. (**a**) The process of conversion; (**b**) the selection of the reference dots for the local scale.

For reducing error effects caused by the nonlinearity of the image, a segmentation method is used to calculate the vertical distance *d,* as presented in Figure 6. The segmentation method refers to dividing *d* into segments $d_{i}$ and calculates $d_{i}$ by using its local scale $s_{i}$ and corresponding $d_{pixel,i}$. Thus, the nonlinear error effects of images can be reduced. In the method, *d* is expressed as $d=\sum_{i=1}^{n}d_{i}$, where *n* is the number of divided segments. For example, *n* is equal to 3 in Figure 6a. As illustrated in Figure 6a, in the pixel coordinates, the pixel length $d_{pixel}$ is divided by the corresponding segments. Each $d_{pixel,i}$ is formed by two points, which can be two reference dots or one reference dot and one laser spot (see in Figure 6a). Moreover, the two reference dots that are the nearest to the segment $d_{pixel,i}$ are used to calculate the corresponding local scale $s_{i}$ by using Equation (17). For example, the reference dots selected in Figure 6b are used to calculate the local scale $s_{i}$ for $d_{pixel,i}$ in Figure 6a as follows:

(17) $$s_{i}=\frac{d_{dot}}{d_{dotp,i}},$$

In Equation (17), $d_{dot}$ is the actual center distance of two adjacent reference dots in the calibration board, which can be obtained by the specification of the board, and $d_{dotp,i}$ is the corresponding pixel length for $d_{dot}$. Once the pixel distance $d_{pixel,i}$ and local scale $s_{i}$ have been obtained for each segment, the vertical distance *d* can be calculated accurately via Equation (18).

(18) $$d=\sum_{i=1}^{n}d_{i}=\sum_{i=1}^{n}s_{i}d_{pixel,i},$$

The distance *L* is measured by the laser rangefinder. To reach a measurement accuracy of the millimeter level [38,39], the phase method based laser rangefinder, which measures the distance by measuring the phase shift between the transmitted and received signals, is recommended to be used. Moreover, to accurately measure *L*, it is necessary to align the optical center of the laser rangefinder, which can be known from the instruction manual, with rotation axis center B of the rotation platform (see Figure 3b). 

Thus, the actual relative angle $\Delta \theta_{act}$ of the rotation platform can be calculated via Equation (19), and the key parameter $A_{z,off}$ can be calculated by Equation (13).

(19) $$\Delta \theta_{act}=\tan^{-1}\frac{d}{L},$$

### 2.4. Measurement Uncertainty

Measurement results are complete only when they are accompanied by a statement of measurement uncertainty. The measurement uncertainty can be estimated using statistical analysis and other information on the measurement process. Since the measurement uncertainty is related to measurement instruments, the sample being measured, the environment, the operator, and other sources, it indicates the quality of measurement results. Therefore, it is necessary to calculate the measurement uncertainty for the measurements that use the proposed calibration technique.

Based on the theory of uncertainty, the relative uncertainty $E_{\Delta \theta_{act}}$ of the actual relative angle $\Delta \theta_{act}$ (calibrated by Equation (11)) can be calculated as follows:

(20) $$E_{\Delta \theta_{act}}=\sqrt{{\left(\frac{u_{A_{z,off}}}{1000\;mg}\right)}^{2}+{\left(\frac{u_{\Delta \theta_{err}}}{\Delta \theta_{err}}\right)}^{2}},$$

where $u_{\Delta \theta_{err}}$ is the uncertainty of the raw relative angle obtained in practice; $u_{A_{z,off}}$ is the uncertainty of $A_{z,off}$ obtained from the calibration technique, which can be calculated as follows:

(21) $$u_{A_{z,err}}=1000\;mg\sqrt{{\left(\frac{Lu_{d}}{\left(L^{2}+d^{2}\right)\tan^{-1}\frac{d}{L}}\right)}^{2}+{\left(\frac{du_{L}}{\left(L^{2}+d^{2}\right)\tan^{-1}\frac{d}{L}}\right)}^{2}+{\left(\frac{u_{\Delta \theta_{err}}}{\Delta \theta_{err}}\right)}^{2}},$$

where $u_{d}$ is the uncertainty of vertical movement *d*; $u_{L}$ is the uncertainty of distance *L* and $u_{\Delta \theta_{err}}$ is the uncertainty of the raw relative angle containing errors, which is measured in the calibration procedure. The values of parameter $u_{d}$ and parameter $u_{L}$ are the same for all the MEMS inclinometers mounted on the same rotation platform, while the raw relative angles $\Delta \theta_{err}$ and calibration parameters $A_{z,off}$ of each MEMS inclinometer are not the same.

**Figure 7:**
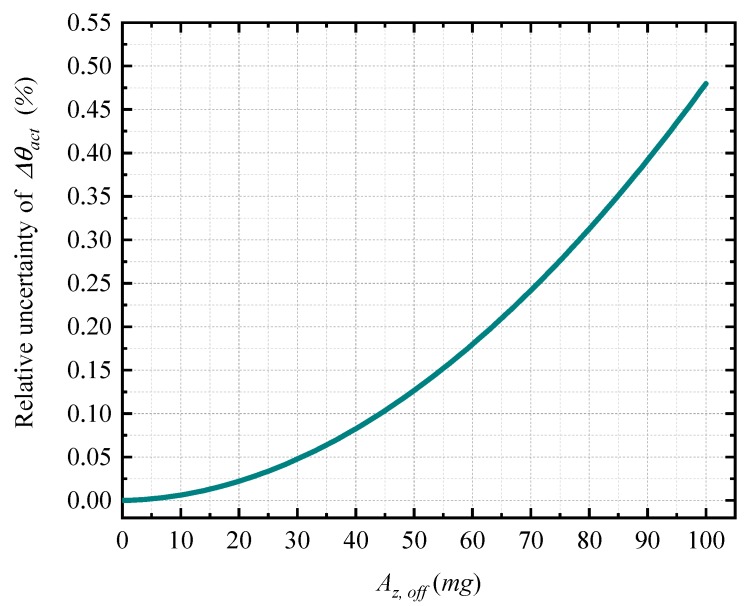
The approximation effect on the uncertainty of $\Delta \theta_{act}$.

The single-parameter calibration model is based on the approximate method, so it is necessary to analyze the effect of the approximation method on the uncertainty of relative angles $\Delta \theta_{act}$. The effect of the approximation relates to the measurement range and the key parameter $A_{z,off}$. The measurement ranges of −5 to +5° satisfies the requirement of structural deformation measurement. Thus, in this paper, the effect of the approximation method is analyzed in this range. In Figure 7, it shows the maximum effect of approximation on the relative uncertainty of $\Delta \theta_{act}$, which is under different values of $A_{z,off}$. For example, to the typical value of $A_{z,off}$ (30 mg) and within the range of −5 to +5°, the maximum relative uncertainty of $\Delta \theta_{act}$, which caused by the approximation method, is 0.05%. 

## 3. Experimental Validation

To evaluate the feasibility of the improved calibration technique, we designed a validation experiment. This experiment composed of two parts: In the first part, the proposed technique was used to calibrate an ADXL355-based MEMS inclinometer. In the second part, the performance of the proposed technique was compared with other methods via a comparison experiment. 

### 3.1. Experimental Setup

**Figure 8:**
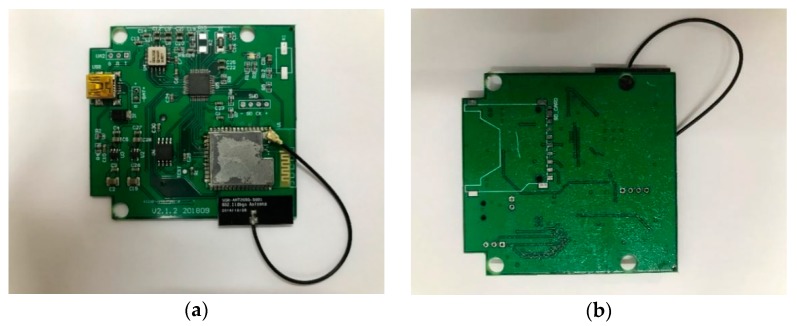
The ADXL355-based MEMS inclinometer. (**a**) The top side; (**b**) the bottom side.

A MEMS accelerometer, namely, ADXL355, is used for the sensing module of the MEMS inclinometer in this validation experiment. The ADXL355 is a low-noise-density 3-axis MEMS accelerometer. Its industry-leading long-term stability enables satisfactory performance in angle measurements. Moreover, due to its low power consumption, ADXL355 is suitable for wireless systems in structural health monitoring [40]. Figure 8 shows a prototype of the MEMS inclinometer. Table 2 presents its specifications. 

In the first part of the validation experiment, we used an NTS-322R4 total station, a Nikon digital single-lens reflex (DSLR) camera, a UT390G laser rangefinder, and a dot calibration board to obtain the key parameter $A_{z,off}$ for the ADXL355-based MEMS inclinometer. The laser transmitter of the NTS-322R4 total station is used to project a laser spot on the calibration board. It is noted that the total station is served as a rotation platform. The specifications of the devices are presented in Table 3, Table 4, Table 5 and Table 6. 

**Figure 9:**
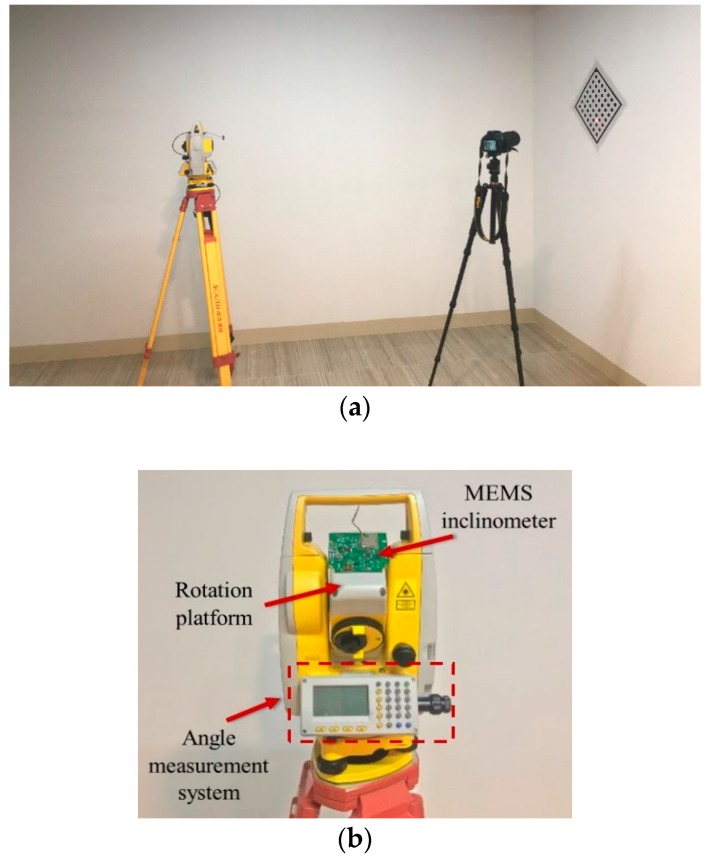

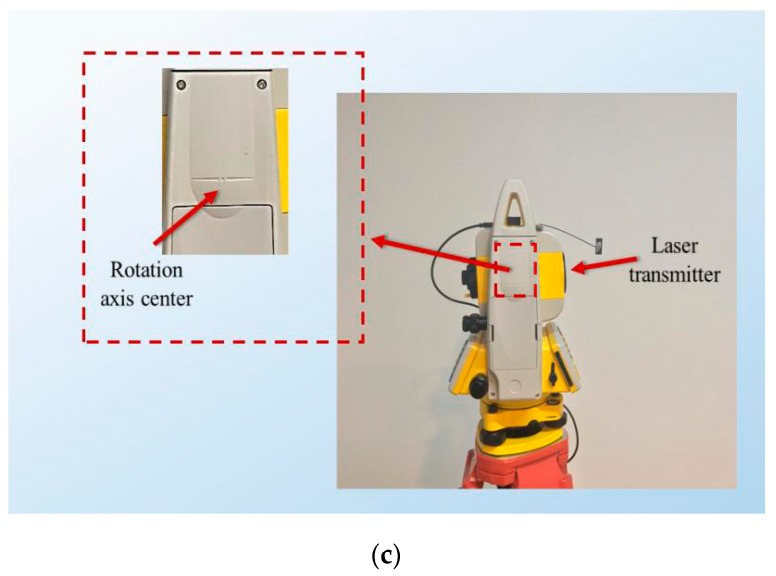
Setups for the validation experiments. (**a**) The setup for the calibration experiment; (**b**) the setup of the devices on the rotation platform; (**c**) the rotation axis center of the rotation platform.

The calibration setup for the ADXL355-based MEMS inclinometer is shown in Figure 9. The MEMS inclinometer was glued to the top of the rotation platform. For the NTS-322R4 total station, the laser transmitter is mounted to the rotation platform and the optical center of the laser transmitter is well aligned with the rotation axis center. For measuring parameter *d*, the camera was setup on a separate tripod, which kept it stationary during the experiment, and the dot calibration board, which has an accuracy of ±0.005 mm, was attached to the wall stably. Thus, in the experiments, the image processing method can achieve an accuracy of ±0.05 mm for measuring the parameter *d*. To measure the parameter *L* accurately, the optical center of the UT390G laser rangefinder was aligned with the rotation axis center of the rotation platform (See Figure 9c). The UT390G laser rangefinder is based on the phase method and has a measurement accuracy of ±1.5 mm. The measurement data were acquired via the wireless transmission module to the computer. The calibration procedure is as follows:

Step 1: Use the laser rangefinder to measure the distance *L.* Collect the initial raw angle measurement values of the ADXL355-based MEMS inclinometer and use the camera to record the initial image which has the laser spot. 

Step 2: Rotate the platform and then collect the changed raw angle values and record the image.

Step 3: Repeat Step 2 and collect the data. 

Finally, several groups of data about parameters *d* and $\Delta \theta_{err}$ are obtained, and the key parameter $A_{z,bias}$ can be calculated. 

After completing the calibration of the ADXL355-based MEMS inclinometer, a comparison experiment was conducted to evaluate the performance of the proposed technique. Since the NTS-322R4 total station has a high-accuracy angle measurement system, the experiment was conducted on the rotation platform of the total station, as shown in Figure 9b. The angle measurement system is composed of an electronic theodolite, in which the resolution is 0.0002° and the accuracy is ±0.0005°. Thus, the measured angle of the angle measurement system is referred to as the reference relative angle. In the experiment, the proposed technique was also compared with the traditional six-parameter method [21]. 

The six-parameter method is a calibration method recommended by manufacturers for low-cost MEMS accelerometers, and its calibration equations are expressed as follows:

(22) $$A_{off}=0.5\times \left(A_{+1g}+A_{-1g}\right),$$

(23) $$Gain=0.5\times \left(\frac{A_{+1g}-A_{-1g}}{1000\;mg}\right),$$

(24) $$A_{act}=\frac{A_{ot}-A_{off}}{Gain},$$

where $A_{+1g}$ and $A_{-1g}$ are the acceleration output values of the axis to be calibrated, which are placed under the +1 g and −1 g field, respectively. $A_{off}$ is the offset of the axis; Gain is the scale factor of the axis; $A_{act}$ is the actual acceleration output value of the axis; $A_{ot}$ is the raw output value of the axis, which contains errors. The units of $A_{act}$ and $A_{ot}$ are mg.

The comparison experiment was conducted as follows:

Step 1: Collect the initial raw angle measurement values of the ADXL355-based MEMS inclinometer and the reference angle values from the angle measurement system of the total station.

Step 2: Rotate the platform and collect the changed raw angle values of the MEMS inclinometer and the changed reference angle values of the angle measurement system.

Step 3: Repeat Step 2 and collect the data.

This experiment has two increments. The first increment was 0.5°, which was applied to the measurements from −3 to 3°. The second increment was 1°, which employed in the ranges from −5 to −3° and from 3 to 5°.

### 3.2. Results and Discussion

#### 3.2.1. Angle Calibration Experiment

Table 7 presents the results of the calibration experiment. We collected four groups of data. The distance *L* is 3897.0 mm. The movement *d* of the laser spot varies from 107.78 to 279.01 mm. The data of raw relative angles $\Delta \theta_{err}$ range from 1.605 to 4.146°. By using these measured values, the values of the parameter $A_{z,off}$ and the corresponding measurement uncertainty have been calculated. In the four selected groups’ results, the average value of $A_{z,off}$ is −12.6 mg, and its standard deviation is only 0.4 mg, which accounts for 3.11% of the average value. The average value of $A_{z,off}$ is used for calibration, which has an uncertainty $u_{A_{z,err}}$ of 0.4 mg. Based on the average value of $A_{z,off}$, the relative uncertainty $E_{\Delta \theta_{act}}$ of the calibrated actual relative angle $\Delta \theta_{act}$ can be obtained by Equation (20), which is 0.06%.

**Figure 10:**
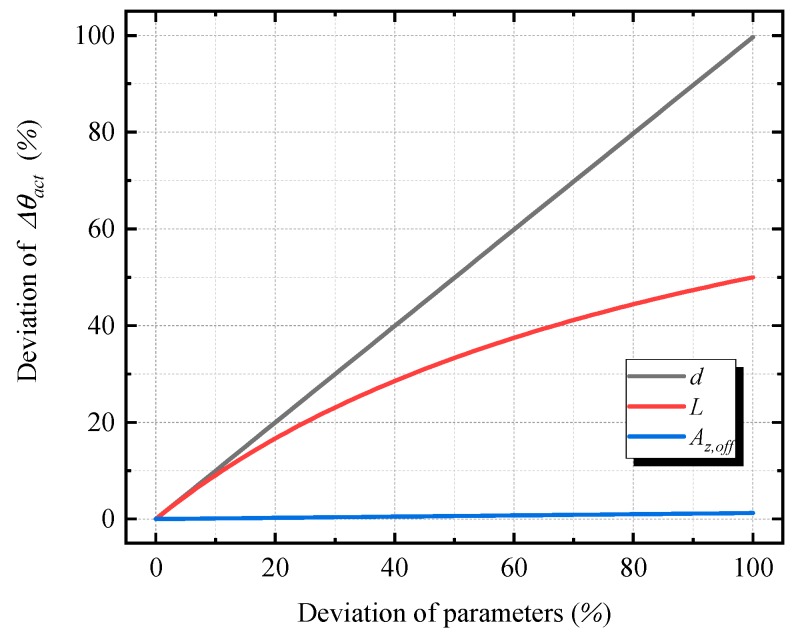
The deviation effects of parameters (*d*, *L*, and $A_{z,off}$) on the calibrated relative angle.

For evaluating the feasibility of the proposed method, it is necessary to analyze the possible error effects on the calibrated relative angles $\Delta \theta_{act}$, which are caused by the deviations of parameters (*d, L* and $A_{z,off}$). As shown in Figure 10, the deviation of parameters *d* and *L* have significant effects on the calibration results. Thus, to obtain reliable results, it is necessary to analyze the magnitude of the deviation of the parameters.

The error from the deviation of distance *L* is minor because the deviation is much smaller than the magnitude of the distance *L*, and the error effect decreases with the distance *L.* By increasing the calibration distance, this error effect can be reduced significantly. 

The errors of the vertical movement *d* contribute substantially to the errors of calibrated relative angles. The magnitude of the errors of *d* depends on the dot calibration board and the image processing algorithm. First, the lattice center actual distance $d_{dot}$ on the dot calibration board is used as the reference to calculate the scale *s* (the ratio of the actual length to the pixel length) and the actual movement *d*. Since the maximum error of $d_{dot}$ is less than 0.005 mm on commonly used calibration board, the error from the dot calibration board accounts for only 0.01% of the value *d* (*d* is equal to 40 mm in the experiment). 

The errors of the image processing algorithm originate from the detection process and the nonlinearity of the scale *s* of the conversion process. The accuracy of detection depends on the shapes of the dot and the laser spot. Round shapes in the experiments are conducive to precise detection. In addition, since the movement distance *d* is small, the laser spot shape is almost identical after moving. Thus, detection before and after moving will correspond to the same point of the spot, according to Equations (14) and (15). Therefore, the error that originates from the detection algorithm is negligible. The nonlinearity of the scale distribution can be resolved via the calibration of the camera. In this paper, since the calibration board is provided as the reference, the local scale of interest can be calculated from the actual lattice center distance $d_{dot}$ and the corresponding pixel distance $d_{dotp,i}$, as expressed in Equations (16) and (17). By using the local scale, the accuracy of the distance *d* can be substantially improved. As shown in Figure 10, with the same deviation degree of *d* and *L,* the deviation of *d* causes a larger deviation of the calibrated results $\Delta \theta_{act}$. Thus, a precise image processing algorithm could significantly reduce the errors of the calibrated relative angles.

In addition, according to Equation (11), a 1% deviation of $A_{z,off}$ will cause approximately 0.001% deviations of the calibrated relative angles $\Delta \theta_{act}$. Since $A_{z,off}$ has a 3.11% deviation in the calibration experiment, the possible deviation of $\Delta \theta_{act}$ caused by $A_{z,off}$ is only 0.03%. Therefore, the proposed calibration technique is feasible for the calibration of MEMS inclinometers for civil structures. 

#### 3.2.2. Comparison Experiment

In the comparison experiment, we compared the six-parameter calibration method with the improved technique. We placed the ADXL355-based MEMS inclinometer in six positions to obtain the corresponding six parameters, as listed in Table 8. The offset of the *z*-axis is −12.69 mg, which is almost the same as the value of $A_{z,off}$ that is calculated in Table 7. Since the offset mainly causes the error of raw relative angles $\Delta \theta_{err}$, it is reasonable that the parameter $A_{z,off}$ and the offset of the *z*-axis obtained by the six-parameter method are close in value. This result demonstrates that the proposed calibration model develops from the traditional calibration models by simplifying the redundant parameters.

In the comparison experiment, four types of results are presented, which are based on four corresponding methods. The first type is the relative angle value prior to calibration, which is referred to as the raw relative angle in the following discussions. The second type is the value that is calibrated via the improved technique, which is referred to as the improved relative angle. The third type is the value that is calibrated via the six-parameter method, which is referred to as the six-parameter relative angle. The fourth type is the value that is measured via the angle measurement system of the total station, which is referred to as the reference relative angle. The value of the fourth type is regarded as the reference value, which has an accuracy of ± 0.0005° (i.e., the reference value is theoretically better than the other three types of results.).

**Figure 11:**
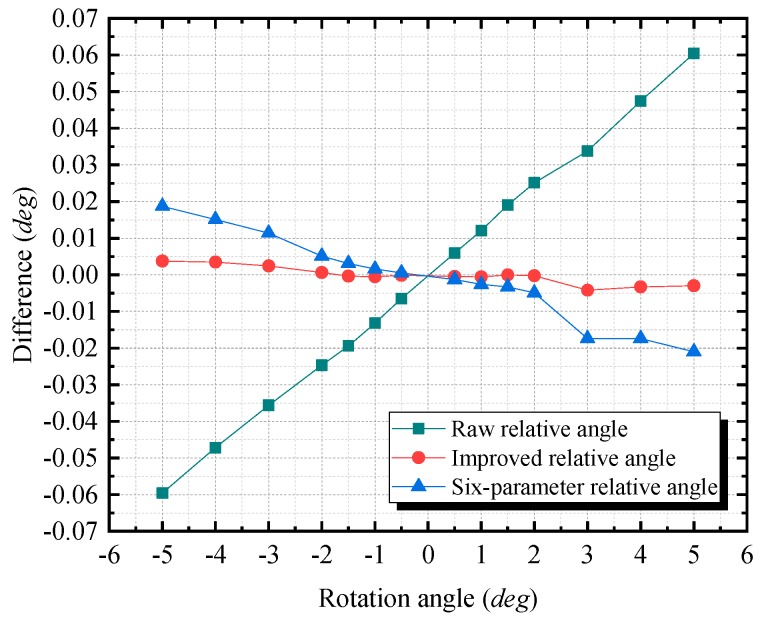
The differences of the measured angles (the raw relative angle, the improved relative angle, and the six-parameter relative angle) from the reference relative angle. The difference is equal to the value measured by different methods minus reference relative angle.

In Figure 11, the differences of the raw relative angle and of the six-parameter angle (the difference is equal to the value measured by different methods minus reference relative angle) change linearly as the rotation angle increases from −5 to 5°. The raw relative angle has a maximum absolute difference of 0.060° at 5°, and the six-parameter relative angle has a maximum absolute difference of 0.019° at −5°. For the improved relative angle, its differences stably lie along the *x*-axis and it has a much smaller maximum absolute difference of 0.004° corresponds to 3°. This maximum absolute difference accounts for only 6.95% of the raw relative angle’s maximum absolute difference, and for 22.46% of the six-parameter relative angle’s maximum absolute difference.

**Figure 12:**
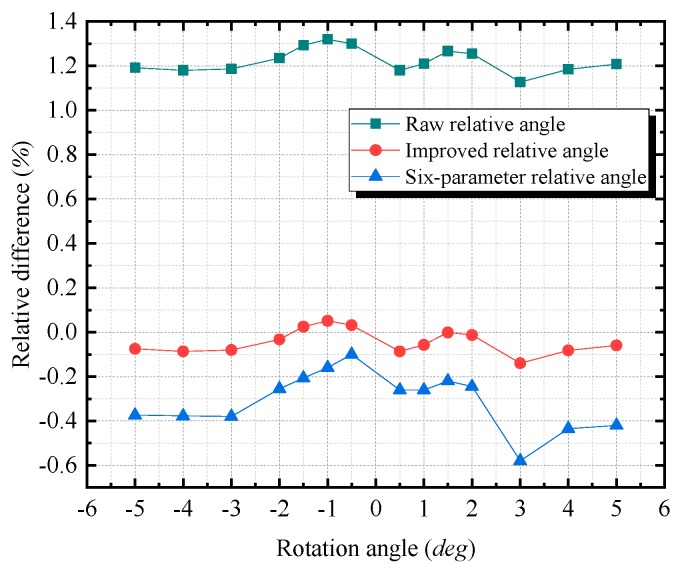
The relative differences of the measured angles (the raw relative angle, the improved relative angle, and the six-parameter relative angle) from the reference relative angle. The relative difference is equal to (value measured by different methods – reference relative angle)/reference relative angle.

In Figure 12, the lines that correspond to the relative differences (the relative difference is equal to (value measured by different method—reference relative angle)/reference relative angle) of the first three types resemble horizontal lines, although the line of the six-parameter relative angle exhibits slight fluctuations. This horizontal tendency demonstrates that the relative differences of the first three types could be constant. The relative differences are approximately 1.22% for the raw relative angle, approximately −0.04% for the improved relative angle, and approximately −0.31% for the six-parameter relative angle. Furthermore, the results demonstrate that the angle measurement errors are caused by a scale factor. For the improved calibration technique proposed in this paper, it reduces the scale factor error effect via the approach as that expressed in Equations (11) and (12).

The reason for the lower accuracy of the six-parameter method is that the number of placed positions is small. In our case, six positions are used. Although satisfactory accuracy in the measurement range of interest can be realized by placing more positions around this range, this is a time-consuming procedure, and expensive devices would be required. The improved calibration technique is both simple and has satisfactory accuracy. Thus, it is a potential calibration method for the MEMS inclinometer for civil structures.

## 4. Application

### 4.1. Experimental Setup

**Figure 13:**
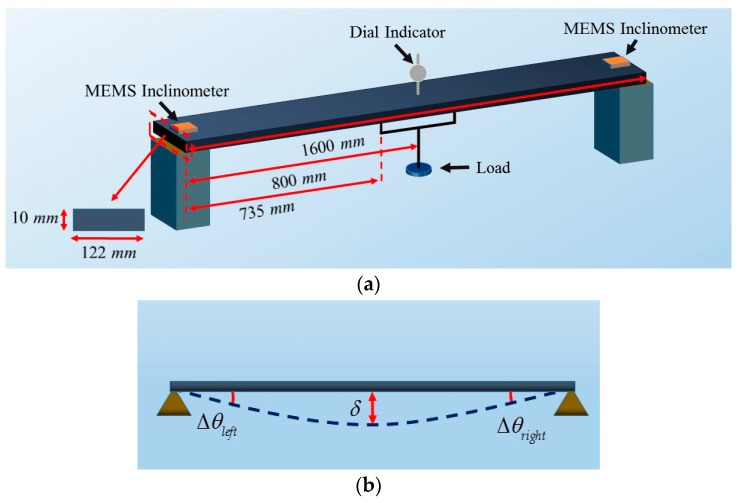

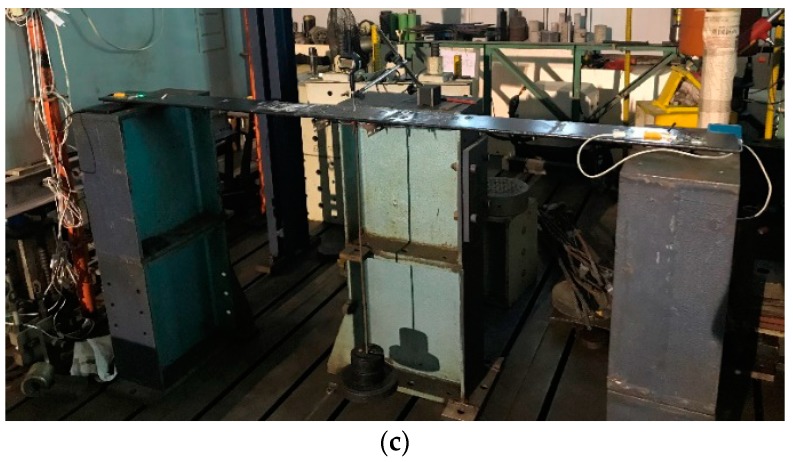
The setup for the deflection experiment. (**a**) A schematic diagram of the experimental setup; (**b**) an illustration of the δ which is measured in the experiment; (**c**) the front view of the setup.

To evaluate the performance of the calibrated MEMS inclinometer, we applied it to a deflection measurement experiment on a scale simply supported beam model. As shown in Figure 13, two MEMS inclinometers were glued on the ends of the beam, and a dial indicator was installed in the middle of the beam. A loading platform was set in the middle of the beam, and the loads were applied symmetrically to the beam, as illustrated in Figure 13c. The vertical deflection *δ* (as illustrated in Figure 13b) was generated by adding loads. In the experiments, there were three loading increments, namely, 1, 5, and 10 kg, which aimed at producing various deflection changes. The first increment was used in the loading range from 1 to 10 kg, the second increment was employed in the range from 10 to 30 kg, and the third increment was applied in the range from 30 to 50 kg.

In the experiment, five types of deflection measurement values are obtained for the midpoint of the beam. The first type, namely, $\delta_{rfdf}$, is measured by the dial indicator directly, which is referred to as the reference deflection. This type is used as a reference since it has an accuracy of ±0.001 mm, which is theoretically better than other types of results. The second type, namely, $\delta_{rdf}$, is calculated from the angles prior to calibration, which called the raw deflection. The third type, namely, $\delta_{idf}$, is obtained from the angles that were calibrated via the improved calibration technique, which is referred to as the improved deflection. The fourth type, namely, $\delta_{spdf}$, is obtained via the six-parameter method, which is referred to as the six-parameter deflection. The values of the raw deflection, improved deflection, and six-parameter deflection are calculated based on the conversion relationship between the relative angles at the end of the beam and the deflections in the midpoint of the beam. This relationship can be expressed as Equation (25) [41], which comes from the classical theories of simply supported beams.

(25) $$\delta =\frac{2\Delta \theta \left(l^{2}+lb-0.5b^{2}\right)}{3\left(l+b\right)},$$

Theoretically, $\Delta \theta_{left}$ and $\Delta \theta_{right}$, as illustrated in Figure 13b, should be equal. However, since the ideal symmetric loading is difficult to realize and materials of the model are uneven, $\Delta \theta_{left}$ and $\Delta \theta_{right}$ are unequal. In the experiment, we used the average value of $\Delta \theta_{left}$ and $\Delta \theta_{right}$ for Δθ of Equation (25). In Equation (25), *l* is equal to 800 mm, which is half the length of the beam; *b* is the distance between the loading point on the beam and the midpoint of the beam. *b* is 65 mm, which is equal to 800 minus 735 mm in Figure 13a; and δ is the deflection on the midpoint of the beam, which is expressed in mm. 

The fifth type, namely, $\delta_{tdf}$, is calculated via the theoretical formula, which is referred to as the theoretical deflection, as follows:

(26) $$\delta_{tdf}=\frac{F_{load}\left(l-b\right)\left(l^{2}+2bl-b^{2}\right)}{6EI},$$

This formula is based on the relationship between the load and the corresponding deflection. In Equation (26), *EI* is equal to $2.24\times 10^{9}\;N\cdot mm$, which is the bending stiffness of the beam; $F_{load}$ is the load, which is expressed in *N*; and $\delta_{tdf}$ is the deflection of the middle of the beam, which is expressed in mm.

### 4.2. Results and Discussion

**Figure 14:**
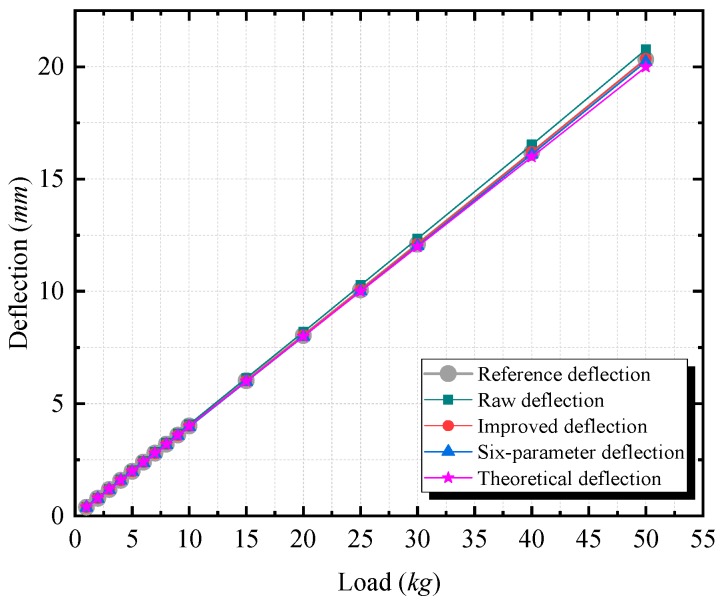
The deflection measurement results of the reference deflection, the raw deflection, the improved deflection, the six-parameter deflection, and the theoretical deflection at the same loads.

**Figure 15:**
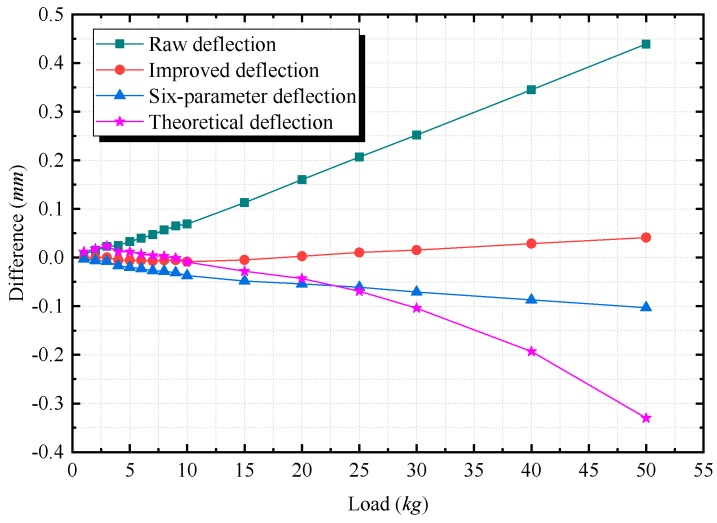
The differences of the raw deflection, the improved deflection, the six-parameter deflection, and the theoretical deflection from the reference deflection. The difference is equal to the value measured by different methods minus reference deflection.

According to Figure 14, the measured deflections vary from 0 to 20.5 mm. The lines that correspond to the five types of values all show linear tendencies. As the loading increases, the difference (the difference is equal to the value measured by different methods minus the reference deflection) of the improved deflection remains small, as shown in Figure 15. The differences of the raw deflection, the improved deflection, and the six-parameter deflection are all linearly related to the load and increase as the loading increases. In Figure 15, the slope of the raw deflection is the steepest, and that of the improved deflection is the smallest. The difference of the theoretical deflection changes nonlinearly, which results in the largest absolute difference among all types. The maximum absolute difference is 0.439 mm for the raw deflection and is 0.330 mm for the six-parameter deflection. Via the proposed technique, the improved deflection has higher accuracy, with the maximum absolute difference of only 0.042 mm. Moreover, compared with the values without calibration, the accuracy increases by 90.43% using the improved technique, and by 24.83% using the six-parameter method. The comparison shows that after calibration, more accurate deflection measurement values can be obtained. 

**Figure 16:**
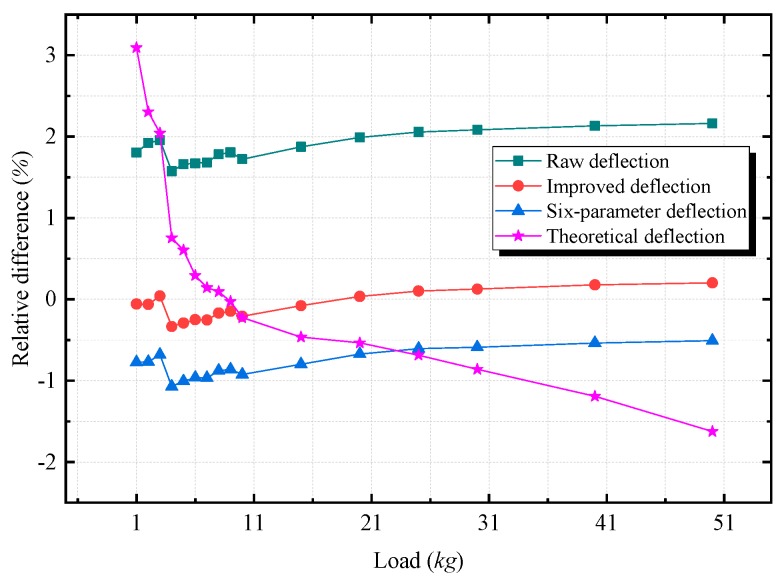
The relative differences of the raw deflection, the improved deflection, the six-parameter deflection, and the theoretical deflection from the reference deflection. The relative difference is equal to (value measured be different methods – reference deflection)/reference deflection.

The relative differences (the relative difference is equal to (value measured by different methods – reference deflection)/reference deflection) of the raw deflection, the improved deflection, the six-parameter deflection, and the theoretical deflection are plotted in Figure 16. The relative differences of the theoretical deflection change nonlinearly due to the inaccurate bending stiffness parameter. Although the relative differences of the raw deflection, the improved deflection, and the six-parameter deflection increase slightly as the load increases, they still resemble horizontal lines. Comparing with the error tendencies in Figure 11 and Figure 12, the tendencies of the errors of the relative angles accord with the tendencies of the errors of the deflections. Therefore, the deflection errors could mainly cause by errors of the measured angles. Thus, by reducing the scale factor-related error effects of the measured angles, the accuracy of the deflection measurement can be improved. In the experiment, the maximum absolute relative difference of the improved deflection is only 0.33%, while the maximum absolute relative difference of the raw deflection is 2.16%. Thus, the proposed calibration technique can significantly increase the accuracy of the deflection measurement results. 

## 5. Conclusions

In this paper, we proposed an improved calibration technique for MEMS accelerometer-based inclinometers. First, a single-parameter calibration model was constructed for obtaining accurate angles. Then, an image-processing-based method was designed for obtaining the key parameter for the calibration model. An ADXL355 accelerometer-based inclinometer was calibrated to evaluate the feasibility of the technique. The calibration experiment indicates that the proposed image-based method can reliably obtain the key parameter, which ensures the accuracy of the calibration. Furthermore, the results of the comparison experiment demonstrate that the difference between the raw data and the reference values change linearly as the measured angle increases. Therefore, the scale error constitutes most of the error for small angular measurements. Via multiplication by a scale factor that is related to the offset, the proposed calibration model reduces the scale error effect significantly. Experiments were conducted to evaluate the performance of the calibrated MEMS inclinometer. The result suggests that the proposed technique can effectively lead to accurate deformation measurements. 

In summary, the proposed calibration technique has the following advantages: (1) The method is easy to be implemented and only contains one parameter but without losing the accuracy, and (2) the calibration setup is simple, and no complicated instruments are involved. Therefore, the improved calibration technique in this paper is a promising candidate for accurate deformation measurement and can be used in a wide range of areas to obtain high accuracy in practical engineering. 

## Figures and Tables

**Table 1:** Micro-electro-mechanical system (MEMS) accelerometers used in civil engineering [22].

	Device	CXL01LF [23]	CXL02LF [24]	LIS344ALH [25,26]	LIS3L02AL [27,28]	LIS3L02DQ [29,30]
Specification						
Interface		Analog	Analog	Analog	Analog	Digital
Noise-Density ($\mu g/\sqrt{Hz}$)		70	140	50	50	110
Range (g)		±2	±1	±2	±2	±2
Offset Change due to Temperature (mg/°C)		FC ^2^	FC	±0.4	±0.5	±0.8
Sensitivity Change due to Temperature (%/°C)		FC	FC	±0.01	±0.01	±0.02
Cross Axis Sensitivity (%)		FC	FC	±2	±2	±2
Maximum Sensitivity mismatch (%)		5	5	5	5	5
Offset (mg)		15	30	50	60	100
Err ^1^ (deg)		0.860	1.719	2.866	3.440	2.923

^1^ Estimated errors caused by offset. ^2^ FC refers to factory calibrate, and the guidebook does not provide relevant information.

**Table 2:** The specifications of the ADXL355 MEMS accelerometer.

Specification	Value
Interface	Digital
Noise-Density ($\mu g/\sqrt{Hz}$)	25
0 *g* Offset (mg)	±25
Range (g)	±2.048/±4.096/±8.192
ADC	20-bit
Output Data Rate (Hz)	0~4000 Hz

**Table 3:** The specifications of the NTS-322R4 total station (Reflectorless).

Specification	Value
Manufacturer	South Group
Dimensions	160×150×330 mm
Weight	5.2 kg
Max Measurement Distance	400 m
Resolution of Angle Measurement	0.0002°
Accuracy of Angle Measurement	±0.0005°

**Table 4:** The specifications of Nikon digital single-lens reflex (DSLR) camera.

Specification	Value
Sensor Size	23.5×15.6 mm
Sensor Type	CMOS
Effective Pixels	24 megapixels
Max Resolution	6000×4000
Focal Length	18–140 mm

**Table 5:** The specifications of the UT390G laser rangefinder.

Specification	Value
Manufacturer	UNI-T
Accuracy of Distance Measurement	±1.5 mm
Max Measurement Distance	150 m

**Table 6:** The specifications of the dot calibration board.

Specification	Value
Dimensions	400×400×0.18 mm
Lattice	7×7
Center Distance	40 mm
Dot Diameter	20 mm
Accuracy	±0.005 mm

**Table 7:** The parameters *d*, *L*, and $A_{z,off}$ of the calibration experiments.

*L* (mm)	*d* (mm)	$\Delta \theta_{err}(\deg)$	$A_{z,bias}(\mathrm{mg})$	$u_{A_{z,err}}(\mathrm{mg})$	Avg. (mg)	Std. (mg)
3897.0	107.78	1.605	−12.7	0.6	−12.6	0.4
	160.09	2.384	−13.1	0.4		
	216.80	3.224	−12.3	0.3		
	279.01	4.146	−12.3	0.2		

**Table 8:** The parameters of the six-parameter method.

Parameters	Value
Scale Factor of *x*-axis	1.00
Scale Factor of *y*-axis	1.02
Scale Factor of *z*-axis	1.01
Offset of *x*-axis (*mg*)	−3.03
Offset of *y*-axis (*mg*)	−8.90
Offset of *z*-axis (*mg*)	−12.69
